# The Experience of Establishing Data Sharing & Linkage Platforms for Administrative, Research and Community-Service Data

**DOI:** 10.23889/ijpds.v4i1.465

**Published:** 2019-02-13

**Authors:** Kiran Pohar Manhas, Xinjie Cui, Suzanne C Tough

**Affiliations:** 1 Alberta Health Services, Seventh Street Plaza, 14th Floor, North Tower, 10030–107 Street NW, Edmonton, Alberta T5J 3E4, Canada; 2 PolicyWise with Children & Families, 9925 109 St NW, Edmonton, AB T5K 2J8, Canada https://policywise.com/; 3 University of Calgary, Departments of Paediatrics and Community Health Sciences, Faculty of Medicine, 2500 University Drive NW Calgary, AB, Canada T2N 1N4 https://www.ucalgary.ca/stough/about

## Abstract

**Introduction:**

Innovative data platforms (e.g. biobanks, repositories) continually emerge to facilitate data sharing. Extant and emerging data platforms must navigate myriad tensions for successful data sharing and re-use. Two Alberta data platforms navigated such processes and factors regarding administrative, research and nonprofit data: the Child & Youth Data Laboratory (CYDL) and Secondary Analysis to Generate Evidence (SAGE).

**Objectives:**

To clarify the social and policy factors that influenced CYDL and SAGE establishment and implementation, and the relationships, if any, between these factors and data type.

**Methods:**

This paper involves a qualitative secondary analysis of two developmental evaluations on CYDL and SAGE establishment. Six-years post-implementation, the CYDL evaluation entailed document review; website user analysis; interviews (n=30); online stakeholder survey (n=260); and an environmental scan. One-year post implementation, the SAGE evaluation included 15 interviews and document review. We used thematic analysis and comparisons with the literature to identify key factors.

**Results:**

Three (not mutually exclusive) categories of social and policy factors influenced the navigation towards CYDL and SAGE realization: trusting relationships; sustainability amidst readiness; and privacy within social context. For these platforms to be able to manage, link or share data, trust had to be fostered and maintained across multiple, dynamic and intersecting relationships between primary data producers, data subjects, secondary users and institutions. Platform sustainability required capacity building and innovation. Privacy and information sharing evolved culturally and correspondingly for these data platforms, which required constant flexibility and awareness.

**Conclusions:**

This analysis calls for more empirical research on the value of data re-use or the detriment in not re-using data. While the culture of information sharing is progressing towards greater openness and capacity for data sharing and re-use, successful data platforms must advocate, facilitate and mobilize analysis and innovation using data re-use while being cognizant of social and policy influences.

## Introduction

“The value of data lies in their use” [1]. Data analytics is increasingly valued for innovation, precision, and quality improvement [2, 3]. Research funding agencies increasingly mandate data sharing practices, wherein data is made available for re-use (also known as secondary use) by others through controlled ways including techniques of data de-identification, access approval processes, and limits to how and where data re-use occurs [4, 5]. Data sharing is increasingly associated with transparency and accountability. Public, private, research and nonprofit organizations are each becoming more data-focused, data-driven, and interested in data sharing for re-use [2, 4—10]. Data sharing differs from open data initiatives: the latter means data are made wholly accessible, conveniently available, and minimally costly to use [11]. Alongside these data-sharing trend have come innovative data platforms – biobanks, repositories and data-focused laboratories and institutes – to facilitate sharing of sensitive data [12—14].

Data platforms promote transparency and accountability by enabling further analyses, verifications, and results’ refinements [8, 10, 15]. The frequency, diversity, complexity, and novelty of research opportunities increase alongside burgeoning data availability. Cost savings are introduced because of economies of scale benefiting participants, researchers, funders, trainees, and the public [8, 10, 16]. The costs of collecting data become efficient as greater uses for that data can be realized through sharing. Research participants’ contributions and time are efficiently maximized as their contributions can support multiple relevant research projects, while future respondent burdens and research costs are decreased because future participants will not be unnecessarily asked the same questions [7, 17—19].

Tenopir and colleagues surveyed a multinational sample of scientific researchers at two time-points (2009/10 and 2013/14) to capture states of data sharing and re-use [7]. They noted an increase in data-sharing behaviours, willingness to share, and risk perceptions [7]. Persistent recognized barriers to data sharing included concerns with risks of re-using others’ datasets; concerns of potential misinterpretation; the need to publish before releasing data; perceptions that data sharing was unnecessary or impermissible [7]. Further barriers to data sharing and re-use included misunderstandings around data management; lack of metadata and formatting standards; and lack of integration across diverse data repositories [7].

Making data available is not an end in itself, whether through data sharing or open data initiatives [20—22]. We use data to create information, which can then facilitate knowledge. Only when data is used, then the opportunities, learnings and efficiencies associated with data can be realized [22, 23]. Data must first be prepared, promoted, and supported to assist secondary users in recognizing and mobilizing existent data [24]. Then data re-use can occur where someone other than the data collector or originator uses the dataset; this furthers the translation of information to knowledge [22]. The proposed benefits of data platforms or data sharing necessarily follow these two events: data preparation, promotion, and then data re-use.

Many social and policy factors influence extant and emerging data platforms in their success in data sharing and re-use. To support future platforms, this paper will present the experience of two data platforms implemented by PolicyWise for Children and Families (PolicyWise) in Alberta, Canada in navigating these factors: the Child & Youth Data Laboratory (CYDL) and Secondary Analysis to Generate Evidence (SAGE) data and research platform [25, 26].

### The Cases

In 2007, PolicyWise established CYDL through the Alberta Child and Youth Initiative Deputy Ministers to link anonymized administrative data across child- and youth- serving ministry partners responsible for education, health, human services, justice and indigenous issues [27]. This platform involves controlled sharing, and re-use, of administrative data collected during provision of public programs. PolicyWise is a non-governmental organization responsible for housing, linking, and analyzing data [27]. CYDL’s research aims are collaboratively honed with partnering Ministries. CYDL aims to improve child and youth health and social outcomes through integrated information and decision-making [27].

Between 2011 and 2016, PolicyWise developed SAGE through partnership with child-focused research institutes, government, and funders [28]. This partnership intended to build on PolicyWise’s data security, analysis and infrastructure expertise, for the purpose of storing, cleaning, cataloguing, and managing data for research and policy re-use and to address gaps in data sharing in Alberta [28]. Officially launched in fall 2016, SAGE first focused on two data types: research data and data from nonprofits and community service organizations. While CYDL conducted data analysis, the SAGE platform focused on facilitating data re-use through support in centralized data housing, cataloguing and managing.

## Methods

We completed a qualitative secondary analysis of two developmental evaluations around the establishment and implementation of CYDL and SAGE [27—29]. The research questions included (a) what social and policy factors influenced the establishment, development and implementation of CYDL and SAGE; and, (b) what relationship, if any, was between these social and policy factors and data type (particularly administrative, research and nonprofit data)? 

### Evaluation Methods

With little provincial precedent, CYDL was a “pathfinder project” for Alberta [27]. The evaluation examined the first six years of CYDL including its process and outputs for the first series of commissioned projects [27]. The evaluation involved mixed methods: document review; analysis of CYDL website user access; informant interviews with managerial, ministerial and research stakeholders (n=30); online quantitative stakeholder survey (n=260); and an environmental scan on practices, policies and documented challenges on the websites of eight linked-administrative-data platforms in Canada and a few key international centres [27]. The qualitative data from interviews and open-ended survey questions were analyzed into themes using content analysis; the quantitative survey data was analyzed according to frequencies using SPSS.

One year after its launch, a developmental evaluation of SAGE was published [28]. This internally-led review aimed to understand SAGE’s potential outcomes, impact, and most influential features; and, to plan ongoing monitoring and improvement [28]. This evaluation included 15 interviews (6 individuals directly involved in SAGE development; 9 external experts) [28]. The external experts were identified through snowball sampling from the internal SAGE participants; they were recruited by email (9 of 11 contacted participated). Interviews were in-person or by phone and lasted about 60 minutes [28]. A SAGE-initiated literature review was updated and reviewed for the purposes of this developmental evaluation. Two independent reviewers analyzed the interview transcripts and themes were determined through discussion and consensus. 

### Secondary Analysis Methods

In this paper, we share a qualitative secondary analysis of the evaluation reports of CYDL and SAGE to determine the common social and policy factors that influenced the establishment, development and implementation of CYDL and SAGE, and whether any relationship exists between these factors and data type. Two co-authors independently considered the methods, data collection and findings from the two reports. Each co-author grouped findings into common themes, which were discussed to garner consensus on the priority and relationships amongst the cross-cutting themes between CYDL and SAGE developmental evaluations. Disagreements were resolved by the third co-author. The credibility of the analysis is promoted through the use of peer review (amongst co-authors); fidelity to the original themes in the evaluation reports; an audit trail of key decisions during theme development; cross-referencing findings with further CYDL and SAGE reports or presentations as well as the literature on social and policy factors associated with data platforms, data sharing and data governance.

## Results

The processes of developing, establishing and implementing CYDL and SAGE were characterized by three categories of influential social and policy factors: (a) trusting relationships; (b) sustainability amidst readiness; and (c) privacy within social context.

### Trusting Relationships

For both CYDL and SAGE, cultivation of trust and relationships was critical to the establishment and implementation of the data platform. For CYDL, relationships were built across and between diverse ministries to assure data access and appropriate CYDL infrastructure. Originally, deputy ministers conducted much of CYDL’s governance. High-level commitment to, and relationships with, CYDL were well-established. Gaps were noted in the lack of coordination at mid- to lower- levels of government; the inconsistency of ministerial staff turnovers; and the lack of legal-privacy expert involvement. CYDL thereafter established a Legal and Privacy Working Group and a Research Working Group with a greater policy role [27].

Approximately 39% of CYDL stakeholder survey respondents felt that they had not received adequate communication of CYDL’s work, which could diminish ongoing relationships and trust. Where communication was deemed inadequate, stakeholder survey respondents noted those inadequacies generally, in one’s own ministry, between ministries, and noted a lack of governance and processual documentation (e.g. decisions, strategies or next steps) [27].

In the face of ministerial and government turnover, CYDL’s longevity appears connected to ongoing communication and collaboration initiatives between CYDL and government. CYDL enhanced its documentation, frequency of meetings and progress report delivery. Sponsors and champions at multiple levels were critical to CYDL progression from concept to analytic data platform [27]. Integrated knowledge translation is a hallmark with research questions co-created between CYDL and ministerial representatives. This promoted knowledge-user uptake and CYDL accountability around data use. CYDL consistently strove to ensure the relevance of their work to ministry priorities and emerging issues. Such effort sustained the trust needed for CYDL to continue as the only provincial non-governmental data platform housing and linking cross-ministerial administrative data.

PolicyWise leveraged and expanded the relationships it formed in establishing CYDL to garner the support to establish SAGE. Three relationships types were particularly important: those with other data repositories, with data producers (including academics, non-profit organizations and data users), as well as with policymakers/institutions. SAGE developed its deposit agreements and high quality analysis approaches through early work at CYDL and work with academic researchers. Relationship building was critical for bringing data into SAGE, especially from the more data-naïve nonprofit sector [28].

SAGE needed to understand the distinct information needs of each nonprofit organization with which it worked. In the nonprofit sector, data capacities are diverse. Some community-service organizations possess resources, experience, and capacity to collect, manage and share data, while others are not well-versed and often reticent to share or re-use data [3, 11]. SAGE worked with each nonprofit organization individually to determine data capacity and needs. This client-focused approach helped the development of trust and promoted data sharing.

For example, to build relationships and understand capacity in the nonprofit sector, SAGE acted as a central data platform and data expert for six nonprofits servicing vulnerable populations in an urban Albertan area. The nonprofits aimed to examine their collective data to build a composite poverty indicator. This goal did not involve transferring data to SAGE for general sharing purposes. SAGE provided policy, technical, and analytical expertise and acted as intermediary. Acting as a trusted resource facilitated conversations with each organization on their data collection and consent processes and the possibility of eventually depositing appropriate de-identified data into SAGE for future re-use. SAGE used LinkWise, an anonymous data linkage software developed by CYDL, to link the data from these organizations to better understand organization overlap and potential for collaboration. Working on data-producers’ goals facilitated trust and relationship building, which will foster SAGE’s success and longevity.

### Sustainability amidst Stakeholder Readiness

The evaluations of SAGE and CYDL discussed the need for, and challenges to, maintaining platform sustainability. The SAGE evaluation defined sustainability to include techniques for data preservation, cost recovery, and maintaining organizational relevance and presence. SAGE’s initial implementation required a long-term vision of being continually responsive to the evolving needs of researchers and data custodians [28].

SAGE used several strategies to promote sustainability. First, SAGE continues to consider cost recovery options such as cost recuperation for select data preparation or management activities by SAGE staff. Currently, SAGE does not charge data producers or accessors, but for select activities or populations this may be an option. Second, SAGE actively plans how it will meet the growing data sharing and re-use needs as the number of SAGE users and depositors increases. Third, SAGE leverages existing capacity in data management and analysis at PolicyWise through CYDL. SAGE seeks further synergistic opportunities, such as the above-described data intermediary role for six nonprofits seeking to compare their data amongst themselves. Fourth, SAGE collaborates with other emerging or established data platforms to ensure alignment, not overlap, in the data re-use space . Broader trends promote data repository establishment and likely sustainability including the ease of start-up, cheaper storage and technological resources, and better internet access [28].

Readiness for respective roles in data sharing and re-use enterprises appears necessary for all stakeholders including the platform, data producers and data users. Building capacity and training are ways to support such readiness, and thus the need for, and sustainability of, the data platform. SAGE actively advocates for “Secondary Use by Design,” (SUD) (elaborated below), by promoting data management capacity. Data management considerations should originate alongside the proposal. Data producers must be trained in all stages of data management to enable broader sharing and future re-use including appropriate processes for data collection, consent, and data cleaning [28]. SAGE actively trains research and nonprofit sectors, particularly junior researchers and interested nonprofits. Training activities include one-on-one support by SAGE staff when preparing for potential data deposit; overview presentations at university and community sites; commissioning and publishing an ethico-legal report on privacy obligations for Alberta nonprofit organizations [30]; and providing training grants for re-use of current SAGE datasets. User training is critical to sustaining data platforms. The SAGE online presence is being expanded to include training videos, and a blog with informative and relevant material.

SAGE and CYDL have both found the question of fiscal sustainability to be challenging and important, which has required creativity [27, 28]. Historically, data platforms with longer-term financial security are often linked to the routine business functions of large institutions (e.g. the federal government or a faculty). But, institutional links are not indefinite guarantees. CYDL receives funding from the provincial government to conduct its cross-ministerial data analyses. This funding changes as government priorities shift. SAGE pursued grant opportunities, which only provide time-limited support. This ebb-and-flow to funding can be taxing to human resources (time-wise and emotionally) to constantly require value and impact propositions and to justify platform existence [31].

If platforms are focused solely on survival, there is less attention on innovation and growth. Both CYDL and SAGE benefited from initial infrastructure support to enable ongoing research and innovation, while fulfilling platform functions. CYDL and SAGE stakeholders and staff recognized that self-sufficiency of data platforms may arise once data assets are abundant; but the consistency and reliability of external financial support is critical at initial implementation [27, 28].

PolicyWise has turned their focus on grassroots initiatives as another avenue towards financial security. CYDL grassroots initiatives lead to leveraging its technological and resource expertise to facilitate SAGE, and as it is an untapped space where SAGE can strategically fill an unmet need. Another grassroots initiative involves SAGE’s data management work with the poverty-focused nonprofits [28].

Finally, CYDL and SAGE invested greatly into mobilizing the principles and practices of good data governance. PolicyWise recognized that sustainability and good governance were connected; such governance is required of the data platforms, and of relevant organizations in research, nonprofit and public settings [27, 28].

### Privacy within Social Context

Both SAGE and CYDL had to learn and adapt to privacy laws, technological capacities, and social context. Working with identifiable information legally and ethically triggers privacy considerations, provincially, nationally and internationally [10, 32, 33]. CYDL had a different experience related to legal interpretations, in part due to the type of data it was working with and due to the changes in society, technology and culture between the establishment of CYDL and that of SAGE.

Data platforms face recognized challenges to facilitate data sharing and re-use including consent processes, privacy risks, governance, access, and communication [17, 34—41]. Privacy concerns arise when identifying information (e.g. health information, human tissues) is involved and centre on potential misuse, stigma, and intrusion. The future uncertainty surrounding research uses, technological advances, changes to data environment and potential security breaches further these concerns [17, 41]. For SAGE and other research or nonprofit data platforms, demonstrated and effective protective practices include stringent data access criteria; privacy protection undertakings; privacy breach sanctions; formal research-ethics-board relationships; oversight committees; and, mobilizing technology for data security [28, 35, 37, 42—46].

Technological advances supported CYDL in addressing privacy concerns by promoting secure data storage, and by facilitating anonymous data linkage. CYDL adopted ISO standard for data security and became the first-use case of large-scale administrative data linkage for anonymous identity resolution software [27]. Through SAGE, PolicyWise developed a privacy-preserving data linkage tool in-house that promoted ease of use and reduced linkage costs [28].

Motivational, economic and political factors directly influence the culture of information sharing and the interpretation of privacy law. During the initial establishment of CYDL, interpretation of privacy laws were fairly conservative, individually focused and risk averse. The paramount concerns during legal interpretation appeared to be related to risk of privacy breaches harming individuals and fear of data misinterpretation harming public bodies [27]. Such harms could include unwanted disclosure, stigma, initiation of counter legal or policy action, or loss of support or funding.

As CYDL has been implemented and work began to establish SAGE, these fears appeared to give way to a recognition of the risk of not sharing or re-using data and of the culture of information sharing viewing information as power (not weakening) and as the common good [47]. PolicyWise has experienced a shift that is slowly reframing individual or organizational protectionism as stagnate because it stems progress and innovation [27, 28]. The utility-privacy balance is now leaning towards utility during privacy law interpretation. Currently, CYDL cross-ministerial projects are approved more quickly and with greater data access compared to the initial test-case projects [27].

For research data, SAGE faced a significant hurdle to gaining data access due to legal, ethical and historical approaches to consent. Before recent trends promoting data sharing and re-use, most research consent forms included language of utmost privacy protection and data confidentiality delimited to the research team. This consent did not permit data sharing with platforms or other researchers. Although retroactive consent for data sharing and re-use is legally permissible, it is highly infeasible, costly, and likely to be incomplete given participant mobility. Much valuable research data was unavailable to SAGE, which was particularly unfortunate given that SAGE aimed to align with increasing research funders’ mandate to share and re-use data by identifying facilities (such as SAGE) to support that endeavour [28].

Some of the challenges on data sharing and re-use stem from the initial design of data collection, including what to collect and the understanding of the proper use of data. For instance, much data collected for service organizations focus on case management and data is often transactional, which is less powerful in providing insight into client population and systems. Research data collection consent is usually limited to predefined analyses and use. When data sharing and re-use are widely accepted as beneficial for broad public good, effort should be made to facilitate the re-use through SUD, where the data sharing and re-use issues are considered and built into the initial data collection plan or data system development. For example, data collected at service organization should consider the use of this data not only for service transaction but also for program improvement, regional or system level understanding of services, and/or linking to other systems to better monitor clients’ needs and program evaluation. Data collected for research should consider consent for future use if appropriate.

SAGE has, thus, focused on capacity building and advocacy for secondary use by design in academic and nonprofit sectors [28]. Training students and researchers alike about the process and potential benefits of data sharing and re-use coincides with the evolving mandates of institutions, funders and journals promoting data sharing and re-use [4, 5]. Slowly, historical peer-to-peer data sharing amongst colleagues is giving way to broad sharing via data platforms; SAGE (and CYDL) bear witness to this slow but deliberate shift [22, 28, 47].

Initial SAGE experience demonstrates a clear need for building data capacity in nonprofits including appreciation of the possible and permissible nonprofit data uses [3, 11, 28]. SAGE commissioned a legal report that demonstrated that nonprofits face legal uncertainty around their privacy obligations, which leads to confusion and lack of uniformity across organizations [30]. Nonprofit data sharing and re-use is marred by newness and diversity challenges like those in the research sector. Many current nonprofit consent forms do not request permission for data sharing and re-use. The infeasibility of retroactive consent is especially poignant for nonprofits with limited resources. The diversity amongst nonprofits was recognized to characterize data collection, their data-readiness for sharing and re-use, and their capacities for data analysis, data management, and privacy policy planning [28, 30].

## Discussion

The experience of CYDL and SAGE is re-iterated in the literature. First, trust in individuals and organizations involved in data platforms has been recognized as crucial to garner public support [48]. Trust requires transparency [48, 49]. Regarding research data sharing and re-use, empirical research with potential research participants confirms the priority and necessity of trust in data platforms, in researchers collecting or re-using data, and in institutions surrounding the platform. Without this trust, data sources will likely not permit contributions from their data [17, 38, 39, 50]. When asked about their data-sharing practices, relationships between researchers built on trust are leading types of contexts where data sharing and re-use abound [22, 51—53]. The sharing of administrative data is highly bound to political factors including the existence of trust amongst parties [54].

Second, CYDL and SAGE have emphasized good governance and fiscal creativity to promote their sustainability. Organizational and governance issues are rarely discussed in data sharing and re-use contexts [55]. A 2011 literature review found only 33 published scientific papers on data governance, with the first published in 2005 [56]. PolicyWise’s experience herein corroborates, however, extant literature that connects data quality, trust and good governance [55]. Good data governance entails monitoring and evaluation of data policies [57]. Data governance domains include data principles, data quality, metadata, data access, and data lifecycle parameters [58, 59]. When governance policies are explicit, it promotes foresight, prevents challenges and better enables trouble-shooting when challenges appear. Decision-making domains are clearly detailed as are the locus of accountability for decision-making (and the source of resources when facing challenges). For SAGE and CYDL, clear data governance policies enabled individualized allocations of responsibility between data producers, data re-users, and data platform personnel.

Finally, the social, technological and ethical factors surrounding SAGE and CYDL privacy responsibilities and approaches is confirmed in the literature. Data platforms face recognized challenges to facilitate data sharing and re-use including consent processes, privacy risks, governance, access, and communication [17, 34—41]. A systematic review of barriers to sharing public health data (a type of administrative data) (n=65 articles) revealed technical, motivational, economic, political, legal and ethical barriers [54]. CYDL and SAGE considered these barriers whilst approach privacy obligations [27, 28]. CYDL and SAGE aimed to align with best practices in data security and de-identification, which meets the key data governance mechanisms for health information propounded by the Organization for Economic Co-operation and Development (OECD), as well as the original 1980 OECD Fair Information Principles. While these criteria were aimed at health systems, they advocate for the importance of data re-use for public health, research and statistical purposes, and advocate that the health-data processing should include public consultation, accreditation and fair, transparent and independent decision-making around project approvals [33].

Challenges remain for SAGE and CYDL for their continued utility as data platforms. For example, SAGE must recognize the social obstacles associated with academia, including academic competitiveness and lack of recognition career-wise for the efforts of data re-use [7, 20, 22]. Most nonprofits, like researchers, must competitively apply for funding [3, 11]. More widespread advocacy and capacity-building around data sharing and re-use will support greater collaboration amongst researchers, nonprofits and other data producers. Also, it is difficult to empirically measure and link the impact of data sharing and re-use to successes [22], but such evidence could overcome these social-context obstacles. Research has demonstrated the tangible harms in not re-using data towards improvement and innovation [47].

We recognize that there are limitations to this qualitative secondary analysis. First, we did not have access to the primary data collected, but rather the evaluation reports. We were unable to bring the transcription quotes to further support our development of the common themes. Second, the methods of the developmental evaluations were quite distinct, with the CYDL evaluation involving a larger sample and a highly mixed-methods approach. The lack of parity in the primary data collection and analysis may impact the credibility of our common themes due to the difference in thickness of description in the SAGE versus CYDL evaluation. Despite these limitations, we proffer evidence from both evaluation reports that speak to a common experience in the factors and considerations influential to developing data platforms for data sharing and re-use across three distinct data types.

## Conclusion

We share our learnings in establishing two data platforms aimed towards data sharing, linkage and re-use. The learning process necessitated negotiation through three issues: building and maintaining trusting relationships between institutions, primary data producers, data subjects, and secondary users; cultivating sustainability and readiness for the platform and for communities of public, nonprofit and research organizations; and patiently but innovatively evolving interpretations of privacy and information sharing concerns alongside evolving social contexts. CYDL and SAGE have had to be flexible to survive. Data readiness amongst organizations and researchers is growing, which will move data platforms forward.

This paper calls, as others do, for more empirical research on the value of data re-use or the detriment of not re-using data [22, 47]. The culture of information sharing is progressing towards greater openness and capacity for data sharing and re-use. But, the uptake of shared data by re-users in positions to translate learnings into tangible innovations is critical. Researchers and knowledge users must advocate, facilitate and mobilize analysis and innovation using data re-use; academic and nonprofit reward systems must be reframed so that traditional successes in competitive spheres are not forgone when expanding the possibilities of data [7, 20].
